# A Case Report and Literature Review of Prostatic Tuberculosis Masquerading as Prostate Cancer: A Diagnostic Challenge in a Tuberculosis-Endemic Region

**DOI:** 10.3390/tropicalmed10050145

**Published:** 2025-05-21

**Authors:** Yonathan William, Marto Sugiono, Patricia Diana Prasetiyo, Adelbertus Erico, Gilbert Sterling Octavius

**Affiliations:** 1 Department of Radiology, Faculty of Universitas Pelita Harapan, Tangerang 15811, Indonesia; 2 Siloam Hospital Kebon Jeruk, Jakarta 11530, Indonesia; 3 Pathology Anatomy, Faculty of Universitas Pelita Harapan, Tangerang 15811, Indonesia

**Keywords:** prostatic tuberculosis, prostate cancer, magnetic resonance imaging

## Abstract

A male in his 60s presented with a four-month history of dysuria and lower urinary tract symptoms (LUTS). He had a history of elevated PSA and benign prostatic hyperplasia (BPH), previously treated with transurethral resection of the prostate (TURP). Multiparametric MRI (MP-MRI) revealed a PI-RADS 5 lesion, raising suspicion of malignancy. However, histopathological analysis from MRI fusion-targeted biopsies confirmed tuberculous prostatitis. The patient was treated with antituberculosis drugs, resulting in symptomatic improvement and a significant PSA decline. This case highlights the diagnostic challenge of distinguishing tuberculous prostatitis from prostate cancer, particularly in tuberculosis-endemic regions. Our literature review reveals that patients with tuberculous prostatitis undergoing MRI are at least 50 years old, originate from endemic areas, and exhibit PI-RADS scores ranging from 2 to 5, indicating inter-rater variability. Histopathological confirmation remains essential in cases with ambiguous imaging and clinical findings.

## 1. Introduction

According to the World Health Organization estimates, Indonesia is the second highest country in terms of tuberculosis (TB) burden. In 2021, the incidence rate of TB was 354 cases per 100,000 people, with a mortality rate of 52 per 100,000 individuals. Thus, in a country with a population of around 270 million, approximately 140,400 individuals lost their lives to TB [1]. The genitourinary tract is the second most common form of extrapulmonary tuberculosis, with almost 40–50% being prostatic tuberculosis (PTB). However, the nonspecific nature and clinical findings make the diagnosis challenging and difficult to detect, especially in its early phase [2]. This case report aims to present a rare case of prostatic tuberculosis in Indonesia, a country with a high tuberculosis burden, and to highlight the role of MRI in the diagnosis and management of PTB in a patient with a history of prior biopsy and treatment

## 2. Case Report

A male in his 60s presented with a chief complaint of a four-month history of dysuria and lower urinary tract symptoms (LUTS). The pain was more prominent after voiding. Ten years prior, he felt a similar symptom, but it resolved on its own. There were no signs of fever, diabetes mellitus, bladder outlet obstruction, or neurogenic bladder dysfunction. He suffered from no other comorbidities or underwent any previous treatment for any medical conditions. Three months prior, he underwent transurethral resection of the prostate (TURP) at another hospital for the same symptom with a working diagnosis of simple benign prostate hyperplasia (BPH), with a prostate-specific antigen (PSA) level of 14 ng/mL. His peak flow rate was normal. Tissue analysis from the TURP sample revealed granulomatosis. The culture and sensitivity result from the biopsy tissue did not yield any growth. Therefore, the patient was presumed to have tuberculosis and given a fixed-dose regimen of antituberculosis drugs. However, the antituberculosis drug was stopped due to a previous negative interferon-gamma release assay (IGRA) for mycobacterium. The rationalization behind this approach was unclear as the decision was made in a previous hospital. The patient also routinely consumed bisoprolol for his coronary artery disease, for which he underwent percutaneous coronary intervention before. His vitals and routine physical examinations were within normal limits. A digital rectal examination revealed a bilaterally smooth yet firm prostate gland, which puts prostate cancer into the differential diagnosis.

**Figure 1:**
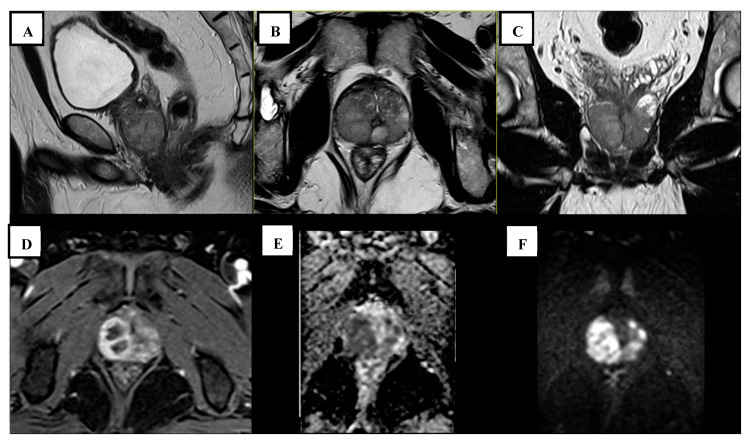
(**A**–**C**) Sagittal, transversal, and coronal T2-weighted turbo spin echo (TSE) showed multiple well-circumscribed homogeneously hyperintense bilateral peripheral zone masses, up to 29 mm in diameter; (**D**) DCE with time-resolved interleaved stochastic trajectory 3D T1-weighted spoiled gradient echo (TWIST-VIBE) showed focal early rim-enhancement; and (**E**,**F**) DWI-ADC with spatially selective parallel transmit excitation diffusion (ZoomIt) showed markedly hypointense ADC (**E**) and markedly hyperintense synthetic b1400 DWI (**F**).

His lab results were notable only for an elevated PSA level of 13.1 ng/mL. Routine blood work and urine tests were normal, and the urine culture showed no bacterial growth. Additionally, his chest X-ray was normal, without any evidence of pulmonary tuberculosis. Prostate MPMRI (Figure 1A–F) showed a mildly enlarged prostate gland, approximately 37 mL in volume. Bilateral peripheral zones were markedly thickened with multiple solid masses, up to 29 mm in diameter, which showed a markedly hypointense signal in ADC, markedly hyperintense signal in high b-value DWI, focal early ring enhancement in DCE, and homogeneously hyperintense in T2WI. According to PI-RADS v2.1, these peripheral zone lesions would be classified as PIRADS 5 due to DWI-ADC findings. However, T2WI and DCE findings are not typical for prostatic cancer; instead, rim-enhancement in DCE is suggestive of an abscess. The study was reported as a possible granulomatous infection with malignancy as a differential diagnosis. There were no molecular tissue diagnostics or cultures performed as the patient refused due to cost constraints.

The patient was recommended to undergo MRI fusion transperineal prostate biopsies to confirm the diagnosis. However, since the patient had just undergone a TURP procedure, he opted for medications first. A regimen of dutasteride and tamsulosin was given, and he was advised to return if the symptoms persisted. Two weeks later, the patient returned for a follow-up and agreed to undergo the biopsy. His symptoms had not improved, and in fact, he went to an emergency department four days before the follow-up since his urinary retention was so severe that an indwelling catheter had to be placed.

**Figure 2:**
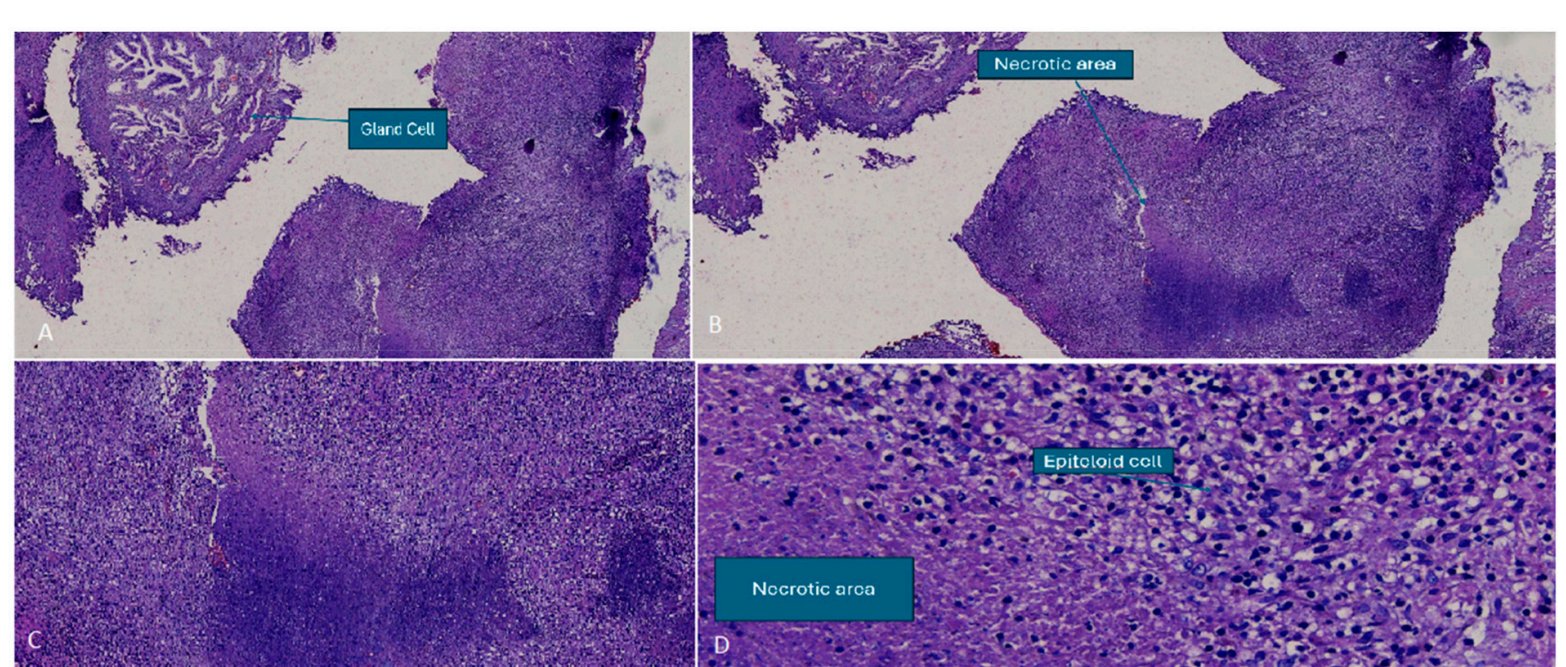
Histological examination—(**A**) epithelial cell proliferation within fibromuscular stroma; (**B**) necrotic region following glandular expansion; (**C**) necrotic area showing inflammatory cell infiltration; and (**D**) epithelioid cells and a necrotic region.

The patient underwent an MRI-targeted transperineal robotic fusion biopsy the following day, which revealed a tubercular granuloma with epithelioid cells, Langhans giant cells, necrotic tissue, chronic inflammatory cells, acinar cells, and basal cell hyperplasia, consistent with a diagnosis of tuberculous prostatitis. Interestingly, the tissue analysis revealed very few areas of necrosis, which contradicts the MRI assumption of abscess formation (Figure 2A–D).

Due to the adverse effects of antituberculosis drugs, the patient was referred to internal medicine. After evaluating the risks and benefits, the patient agreed to continue antituberculosis drugs (ethambutol, isoniazid, pyrazinamide, and rifampicin) separately along with vitamin B6 and cetirizine to control his pruritus.

## 3. Method

A literature review was performed on the sixth of December 2024, searching in PubMed, Google Scholar, and MEDLINE with the keyword “Prostatic Tuberculosis”. Only cases with confirmed results were selected, and the exclusion criteria included those with non-BCG prostatic tuberculosis and cases without MRI findings.

## 4. Discussion

Prostatic tuberculosis is an infective granulomatous prostatitis, commonly occurring alongside tuberculosis of the seminal vesicles and epididymis. PTB is typically asymptomatic or subclinical in its early stages but may later present with nonspecific urinary irritation. This condition is serious and often insidious, frequently discovered incidentally during a partial prostatectomy or biopsy [3]. Since cases are rare and the exact incidence is unknown, another round of biopsy was performed to ensure the diagnosis of PTB.

There are two types of prostatitis tuberculosis: Bacillus Calmette–Guérin (BCG) and non-BCG-related. Prostatic tuberculosis caused by BCG occurs as a rare complication following intravesical BCG therapy for bladder cancer. In contrast, non-BCG prostatic TB typically results from hematogenous spread or direct extension from genitourinary TB. Diagnostically, BCG-related prostatic TB often presents with granulomatous prostatitis on histopathology, with Ziehl–Neelsen staining and mycobacterial cultures frequently negative due to the attenuated nature of BCG [4]. In contrast, non-BCG prostatic TB may demonstrate caseating granulomas with positive cultures or polymerase chain reaction (PCR) for Mycobacterium tuberculosis. Imaging findings on MRI may show prostatitis or prostatic abscesses in both types, but non-BCG TB is more likely to exhibit extensive caseation and calcifications [5,6].

Diagnosing PTB can be challenging, as its symptoms are often subtle and may mimic other diseases, such as prostate cancer. Typically, diagnosis involves a combination of clinical assessment, imaging modalities, and laboratory investigations. While prostate biopsy remains the gold standard, additional tools such as ultrasound, CT scans, MRI, and microbiological testing can provide supportive evidence. In regions where tuberculosis is endemic, such as Indonesia, and particularly when prior histopathological findings indicate tuberculous prostatitis, this differential diagnosis should be carefully considered [7].

A range of microbiological tests is used to confirm the diagnosis, including direct detection of acid-fast bacilli (AFB) through Ziehl–Neelsen (ZN) or auramine staining, culture on selective media, polymerase chain reaction (PCR), and the GeneXpert assay. These examinations are performed on prostatic specimens, such as post-prostatic massage urine, ejaculate, and post-ejaculation urine, as well as urinary samples, including first-void urine collected over three consecutive days, and tissue or pus specimens [8]. However, ZN staining has a low sensitivity and may take up to eight weeks for maximum sensitivity. Furthermore, PCR may not be able to detect whether the infection is currently active or in a latent state [7]. In selected patients with strong clinical suspicion of tuberculosis but negative microbiological results, the diagnosis may still be considered if there is a favorable response to empirical antituberculosis therapy [8]. It is important to note that certain healthcare facilities in Indonesia encounter challenges in sending specimens for laboratory analysis due to transportation issues, long distances, and geographical barriers [9]. Additionally, as test results may take two to eight weeks, there is a risk of patients being lost to follow-up [10].

The rationale for doing another round of biopsy is that tuberculous prostatitis is usually encountered in immunocompromised patients with systemic tuberculosis or those treated with Bacillus Calmette–Guérin infusion for bladder cancer. Thus, the clinical profile still allows for another differential diagnosis, which was prostate adenocarcinoma, due to an abnormally high and persistent PSA level and PI-RADS 5 findings [6]. As mentioned previously, the reference standard for diagnosing PTB is histopathological confirmation through biopsy. In this context, MP-MRI of the prostate plays a crucial role for three key reasons. First, MP-MRI assists clinicians in differentiating PTB from other conditions, such as prostatic adenocarcinoma or prostatic abscess, all of which may present with PI-RADS 5 findings. These conditions require distinct management strategies, making accurate diagnosis essential to minimize morbidity and mortality [11]. Second, lesion localization is critical when a biopsy is indicated. The PI-RADS system provides detailed classification of prostatic regions, enabling urologists to target biopsies more effectively. Inaccurate localization may lead to false-negative results [12]. The discrepancy between MRI suggesting an abscess and the absence of abscess formation on histopathology may be attributed to the biopsy targeting the PI-RADS 5 lesion, rather than the abscess itself, which is typically benign. Finally, in the present case, MRI served as a problem-solving tool, as the patient had previously undergone transurethral resection of the prostate (TURP) and received a short-term antituberculosis regimen, both of which can distort normal prostatic anatomy and obscure classical imaging features.

Differentiating tuberculous prostatitis from adenocarcinoma on MR imaging can be challenging. The appearance of PTB can be nodular or diffuse. The nodular type of prostatic tuberculosis exhibits markedly low signal intensity on T2-weighted imaging, attributed to paramagnetic substances like macrophage-laden oxygen free radicals. On DWI, these lesions show diffusion restriction, while dynamic contrast-enhanced T1-weighted imaging reveals moderate enhancement. In contrast, the diffuse type demonstrates lower T2 signal intensity compared to the normal peripheral zone, though not as low as nodular lesions. Additionally, it presents with diffuse streaky areas of low signal intensity on T2-weighted imaging, known as the “watermelon skin sign”. The diffuse type can be mistaken for prostatic adenocarcinoma [11]. However, tuberculous prostatitis typically has less restricted diffusion and lacks the metabolic peaks, especially citrate level [13], seen in MR spectroscopic imaging, which is more common in large adenocarcinomas. Additionally, tuberculous prostatitis often exhibits less intense enhancement compared to adenocarcinomas. Most patients still undergo a biopsy to rule out adenocarcinoma [6,14]. The introduction of PI-RADS did not help to distinguish between prostate cancer and granulomatous infection. Although some criteria are suggested to differentiate between these two entities, the diagnosis can only be confidently established through histopathologic examination [15].

Our literature review reveals that patients with tuberculous prostatitis who undergo MRI are at least 50 years old and originate from tuberculous-endemic countries [3,16,17,18,19]. None of them suffer from constitutional symptoms, and their PSA levels range from slightly elevated (4.5 ng/mL) [16] to extremely elevated (1768 ng/mL) [18]. The lesions are slightly hyperintense or heterogeneous on T2WI, mostly affecting the transitional zone or the peripheral zone. Interestingly, the PI-RADS score varies from 2 to 5, indicating inter-rater variability in the scoring system [20] (Table 1).

In terms of treatment, BCG-related prostatic TB often resolves spontaneously or with corticosteroids, while non-BCG TB necessitates a standard anti-tubercular therapy regimen, typically consisting of isoniazid, rifampin, pyrazinamide, and ethambutol for at least six months [7]. Surgical intervention, such as transurethral resection or prostatectomy, may be required in severe cases of non-BCG TB with complications such as abscess formation or bladder outlet obstruction [21]. Therefore, distinguishing between BCG-related and non-BCG prostatic TB is crucial for appropriate management and prognosis.

A strong clinical and radiographic suspicion is key to conducting the appropriate diagnostic tests. Most of the literature also reported a PTB incidentally, usually after the histopathologic result suggests granulomatous infections [16,17,22,23]. The standard antituberculosis drug regimen is the preferred treatment option. In cases of urogenital tuberculosis, the duration of treatment varies from 6 to 12 months, and it may be extended based on the clinical response, severity of the disease, and immunocompromised status. Some studies indicate that treatment duration can extend up to two years [24,25].

## 5. Conclusions

In tuberculosis-endemic regions such as Indonesia, PTB should be a key differential diagnosis in patients with elevated PSA and PI-RADS 5 lesions, particularly when clinical findings are atypical for malignancy. This case illustrates the diagnostic challenge posed by PTB, which can closely resemble prostate cancer on both clinical and radiological grounds. Histopathological examination remains essential for definitive diagnosis. Given the inter-reader variability in PI-RADS interpretation and overlapping imaging features, MP-MRI plays a pivotal role in guiding biopsy and clarifying ambiguous findings. Increased clinician awareness, particularly among radiologists and urologists, is vital to avoid misdiagnosis and ensure timely, appropriate management. Additionally, systemic barriers—such as limited access to microbiological testing and loss to follow-up—must be addressed to improve diagnostic accuracy and patient outcomes in resource-limited, high-burden settings.

## Figures and Tables

**Table 1:** Literature review of tuberculous prostatitis mimicking prostate cancer in magnetic resonance imaging.

Author (Year)	Age (Years)	Constitutional Symptoms *	PSA Level (ng/mL)	Previous TURP	PIRADS Score	MRI Findings
Legesse (2024) [18]	69	None	1768	Yes	5	The transitional zone appeared enlarged with mixed signal intensity on T2WI. A focal hypointense lesion was detected in the right basal and midzone of the prostate, extending into the peripheral zone. This lesion was accompanied by capsular obliteration and extraprostatic extension. It exhibited restricted diffusion on DWI and the ADC.
Aslan (2022) [19]	80	None	N/A	No	N/A	A poorly defined, low-signal intensity lesion was identified in the left anterolateral peripheral zone on T2WI. The lesion exhibited restricted diffusion and early ring enhancement.
Li (2023) [3]	52	None	12.4	No	N/A	A mass-like abnormal signal shadow was detected in the peripheral zone, appearing isointense on T1WI, slightly hyperintense on fat-suppressed T2WI, and hyperintense on DWI. Additionally, the lesion had invaded the left seminal vesicle gland, while the left peripheral zone exhibited significant heterogeneous enhancement, and pelvic lymph nodes were observed.
Fonseca (2018) [17]	73	None	6.54	No	2	A small nodule with abnormal diffusion restriction was detected in the left posterior mid-third of the transition zone. It exhibited intense peripheral post-contrast enhancement with a liquefied center, indicative of a microabscess.
Hau (2022) [16]	62	None	4.5	No	3	Multiple lesions were present in the transitional zone and bilateral peripheral zone of the prostate. On T2-weighted imaging, the entire gland’s parenchyma appeared slightly elevated with irregular signals. It showed no early enhancement after contrast administration and exhibited heterogeneous enhancement in the late phase.

PIRADS, prostate imaging-reporting and data system; TURP, transurethral resection of the prostate; MRI, magnetic resonance imaging; PSA, prostate-specific antigen; T1WI, T1-weighted imaging; T2WI, T2-weighted imaging; DWI, diffusion-weighted imaging; ADC, apparent diffusion coefficient; and N/A, not available. * Includes fever, night chills, or weight loss.

## Data Availability

No original data were generated for this manuscript.

## Abbreviations

The following abbreviations are used in this manuscript:

ADC	Apparent Diffusion Coefficient
BCG	Bacillus Calmette–Guérin
BPH	Benign Prostatic Hyperplasia
DCE	Dynamic Contrast-Enhanced
DWI	Diffusion-Weighted Imaging
IGRA	Interferon-Gamma Release Assay
LAMP	Loop-Mediated Isothermal Amplification
LPA	Line Probe Assay
LUTS	Lower Urinary Tract Symptoms
MP-MRI	Multiparametric Magnetic Resonance Imaging
MRI	Magnetic Resonance Imaging
N/A	Not Available
PI-RADS	Prostate Imaging-Reporting and Data System
PCR	Polymerase Chain Reaction
PSA	Prostate-Specific Antigen
PTB	Prostatic Tuberculosis
TB	Tuberculosis
TST	Tuberculin skin testing
T1WI	T1-Weighted Imaging
T2WI	T2-Weighted Imaging
TURP	Transurethral Resection of the Prostate
TWIST-VIBE	Time-Resolved Interleaved Stochastic Trajectory 3D T1-Weighted Spoiled Gradient Echo
